# Identification of age‐specific biomarkers of spinal cord injury: A bioinformatics analysis of young and aged mice models

**DOI:** 10.1002/brb3.3293

**Published:** 2023-11-30

**Authors:** Wei Miao, Zheng Su, Huilin Cheng

**Affiliations:** ^1^ Department of Neurosurgery, Zhongda Hospital, School of Medicine Southeast University Nanjing P. R. China

**Keywords:** age, GSEA, spinal cord injury, WGCNA

## Abstract

**Background:**

Spinal cord injury (SCI) is a debilitating event that often results in long‐term physical damage, disability, and a significant impact on a patient's quality of life. Past research has noted an age‐dependent decline in regeneration post‐SCI. This study seeks to identify potential biomarkers and enriched pathways in young and aged SCI mouse models.

**Methods:**

We retrieved the microarray data of spinal cord samples from SCI mice and control mice from the Gene Expression Omnibus (GEO) database. Differentially expressed genes (DEGs) were identified using the R software and the Linear Models for Microarray Data (limma) package. The Gene Set Enrichment Analysis (GSEA) determined enrichment differences among gene sets. The Weighted Gene Co‐expression Network Analysis (WGCNA) pinpointed clinically significant modules and hub genes in SCI. Kyoto Encyclopedia of Genes and Genomes (KEGG) enrichment analysis was employed to uncover significantly related pathways of crucial genes in SCI.

**Results:**

We found 2560 DEGs in the young SCI group, comprised of 1733 upregulated RNAs and 827 downregulated RNAs. In the aged SCI group, 3048 DEGs were revealed including 1856 upregulated and 1192 downregulated genes. The GSEA revealed 12 enriched signaling pathways in the young SCI group, such as IL6/JAK/STAT3 signaling, interferon alpha response, and interferon gamma response, and 21 signaling pathways in the aged SCI group such as IL6/JAK/STAT3 signaling, E2F targets, and angiogenesis‐related pathways. The WGCNA identified the turquoise module as significantly associated with the clinical traits of both young and aged SCI, and revealed 3181 hub genes. Ultimately, 1858 significant genes in SCI were found, with associated signaling pathways including epithelial‐mesenchymal transition (EMT), interferon gamma response, and KARS signaling.

**Conclusion:**

Our study explored the RNA expression patterns and enriched signaling pathways in young and aged SCI mice. These findings may provide potential biomarkers for targeted SCI therapy.

## Graphical abstract

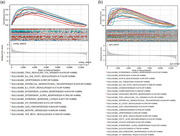

## 1 INTRODUCTION

Spinal cord injury (SCI) is a neurological disease in the central nervous system (CNS) caused by direct or indirect external forces, leading to motor, sensory, and autonomic dysfunction (McDonald & Sadowsky, 2002). Its clinical manifestations are sensory disturbance, dyskinesia, and sphincter dysfunction (McDonald & Sadowsky, 2002). It is divided into primary injury and secondary injury. The initial physical damage to neuronal cell bodies and neuritis mainly occurs in the primary injury, while the subsequent pathological changes in the secondary injury, such as edema, neuroinflammation, metabolic disorder, and local ischemia, may result in apoptosis of neural cells (Profyris et al., 2004). With an estimated mortality rate ranging from 4.4 to 16.7% globally, SCI brings a huge burden to the family and society (Ahuja, Wilson et al., 2017; Jain et al., 2015). Treatment options, such as surgery, stem cell transplantation, gene therapy and drugs, are recommended for SCI patients (Ahuja, Nori et al., 2017; Karsy & Hawryluk, 2019). However, it has been demonstrated that the recovery of the aged population is worse than that of the young population (Wilson et al., 2020), which indicates the significance of exploring potential therapeutic targets in these populations to improve the clinical outcome of SCI therapy.

Age is critically involved in SCI and its repair. In spite of the approximately half cases in adolescents and young adults aged 16 to 30, SCI tends to occur in middle‐aged and elderly patients over 60 years of age (DeVivo & Chen, 2011; Pickett et al., 2006; van den Berg et al., 2010). A study has indicated that 75% of paralyzing SCI patients are distributed in the 40 or over age groups (Geoffroy et al., 2017). Age has been demonstrated to affect axon regeneration post‐CNS injury, and the age‐dependent decline in regeneration may be explained by the reduced *Pten* deletion (Geoffroy et al., 2016). Younger age at SCI is considered a positive predictor, while older age is suggested to exert negative effects on neurological and functional recovery following SCI (Coleman & Geisler, 2004; Oleson et al., 2016; Wilson et al., 2014). Therefore, it is imperative to explore potential biomarkers to better understand SCI pathophysiology and develop novel therapeutic strategies.

It is becoming increasingly clear that biological variations in gene expression may be important molecular phenotypes that influence the pathological process of disease (van Dam et al., 2018). Recent studies in neuroscience have indicated that SCI is a multigene disease and that its pathogenic mechanism is related to alterations in gene expression. Therefore, the identification of associated genes in SCI may offer new insights into gene function along with possible diagnostic and therapeutic targets (Kyritsis et al., 2021). Bioinformatics makes contributions to the exploration of biomarkers in the medical field (Hu et al., 2022; Ren et al., 2017; D. Wu & Wang, 2015). Gene set enrichment analysis (GSEA), developed by Mootha et al. (2003), is a powerful method that determines the statistical significance of a priori defined gene set concordant differences between two biological states (Subramanian et al., 2005). The per‐gene data were aggregated by GESA in a geneset, and small but coordinated changes in genes in the predefined geneset were detected. By assigning ranking scores to all biomolecules in the gene sets, which is stable and interpretable to measure the biological functions of genes (Eklund & Szallasi, 2008), it evaluates the enrichment differences as well as the enriched signaling pathways (Huang da et al., 2009). Weighted gene coexpression network analysis (WGCNA) is commonly used for the construction of co‐expression networks. Clustering genes and forming modules via correlation in gene expression profiles and identifying module‐feature associations contributes to the exploration of hub genes related to disease (Langfelder & Horvath, 2008).

In the current study, we aimed to explore the differentially expressed genes (DEGs) in young and aged mice, the relevant enriched pathways of these DEGs, and the hub genes in the young and aged SCI groups using bioinformatics. The findings of this study may provide clues for the exploration of potential biomarkers in SCI.

## 2 MATERIALS AND METHODS

### 2.1 Data download

The microarray dataset GSE93561 and matched clinical features were downloaded from the Gene Expression Omnibus (GEO) (https://www.ncbi.nlm.nih.gov/geo/query/acc.cgi) database. This dataset was composed of 12 mouse spinal cord samples from 3 young SCI mice, 3 young control mice, 3 aged SCI mice, and 3 aged control mice. The GSE93561 microarray data were retrieved from the GPL1261 platform.

### 2.2 Exploration of differentially expressed RNAs in young or aged SCI groups

Limma analysis was applied to select DEGs in young or aged SCI groups based on the downloaded gene set. Limma is a method based on linear models to screen DEGs (Ritchie et al., 2015). We used the R software Linear Models for Microarray Analysis (limma) package (version 3.40.6) to analyze data from gene expression experiments and compare the DEGs between experimental groups and control groups. False discovery rates (FDRs) < .05 and |fold change | > 2 were set as the cutoff values. The top 50 upregulated or downregulated genes in the young SCI or aged SCI groups were visualized by the “pheatmap” package to create heatmaps or the “ggplot2” package to create volcano plots.

### 2.3 Identification of hallmark pathways

GSEA software (version 3.0) was downloaded from the GSEA website (http://software.broadinstitute.org/gsea/index.jsp). Samples were divided into young SCI and aged SCI groups. The mh.all.v2022.1.Mm.symbols.gmt gene set was obtained from the MSigDB database (https://www.gsea‐msigdb.org/gsea/msigdb/mouse/genesets.jsp?collection=MH) to evaluate the enrichment differences in different gene sets. On the basis of the gene expression pattern and phenotype grouping, the minimum gene set was set as 5, while the maximum gene set was set as 5000. *p* Values of less than .05 and FDR < .25 were regarded as statistically significant (Subramanian et al., 2005).

### 2.4 Identification of significant clinical modules and hub genes through WGCNA

The WGCNA package in R software was applied to build the gene co‐expression network (Langfelder & Horvath, 2008). Modules are gene collections of high topological overlap similarity. The first main component of the module is the module eigengene (ME), which describes the module expression profile. The association of the coefficient of genes with MEs was represented as module membership (MM), which describes the reliability of a gene correlated to a module. The power of *β* = 9 was used as the soft‐threshold value to build the co‐expression network. A topological overlap matrix (TOM) (Liang et al., 2020) was used for adjacency transformation, the network connectivity of a gene was determined as the adjacency sum with all other genes for the network gene ratio, and the calculation of matched dissimilarity (1‐TOM) was also performed. Then, hierarchical clustering was performed to determine modules with a minimum module size equal to 30. A module was clustered with genes with high absolute correlations, and similar modules with an abline equal to 0.25 were merged. Finally, three modules were identified, and the gray module was regarded as the gene set of genes that could not be allocated to any modules. The module‐clinical data correlation was identified, and significant clinical modules were identified. For the selection of hub genes, Pearson's correlation (Jalkanen et al., 2018) was used to measure the connectivity of genes. Genes with high connectivity inside modules and strong association with the specific clinical trait were regarded as hub genes of the modules. The cut‐off criteria were set as |MM| > 0.9, GS > 0.2, and weight > 0.1.

### 2.5 Kyoto Encyclopedia of Genes and Genomes (KEGG) enrichment analysis

KEGG enrichment analysis (Kanehisa et al., 2021) was used to determine significantly related pathways of intersected DEGs and the hub genes screened by the WGCNA with FDR < .1 and *p* < .05 as cut‐off values.

## 3 RESULTS

### 3.1 DEGs in young and aged SCI mice

**FIGURE 1:**
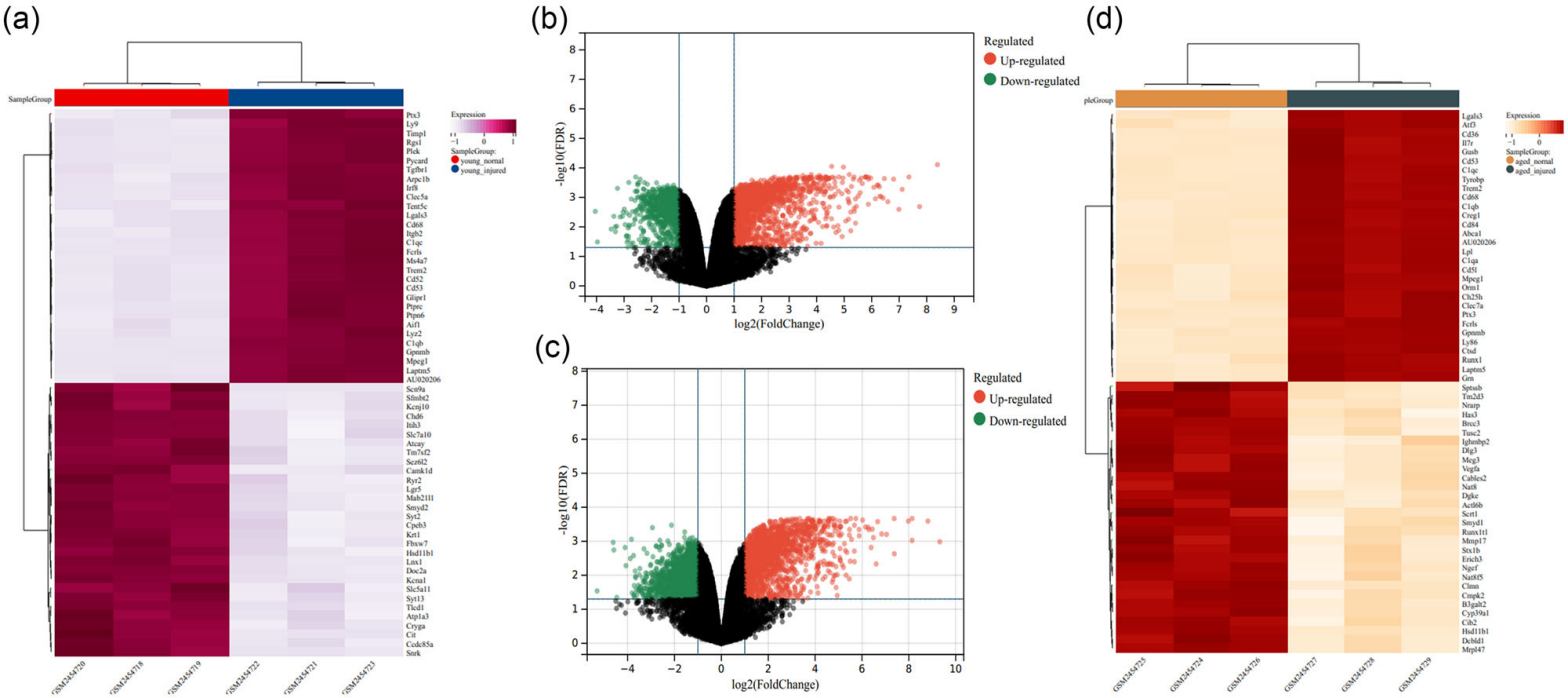
**DEGs in young and aged SCI mice**. (a) Heatmap of the top 50 upregulated and downregulated genes in the spinal cord of young SCI mice compared with the control group. Volcano plot of DEGs in the (b) young SCI mouse group and (c) aged SCI mouse group. (d) Heatmap of the top 50 upregulated and downregulated genes in the spinal cord of aged SCI mice.

The DEGs in the spinal cord of young or aged SCI mice were screened under the conditions of FDR < .05 and |fold change| > 2. The results revealed that there were 2560 DEGs in the young SCI group relative to the control group including 1733 upregulated and 827 downregulated genes. The top 50 upregulated and downregulated RNAs are shown in the heatmap (Figure 1a). The volcano plot also presents the DEGs in young SCI mice, where red indicates upregulated genes and green indicates downregulated genes (Figure 1b). For the aged SCI mice group, a total of 3084 DEGs with 1856 upregulated genes and 1192 downregulated genes were displayed compared with the matched control group. Similarly, these DEGs are shown using a volcano plot and heatmap (top 50 upregulated and downregulated genes) (Figure 1c&d).

### 3.2 Hallmark pathways in the young and aged SCI groups

**FIGURE 2:**
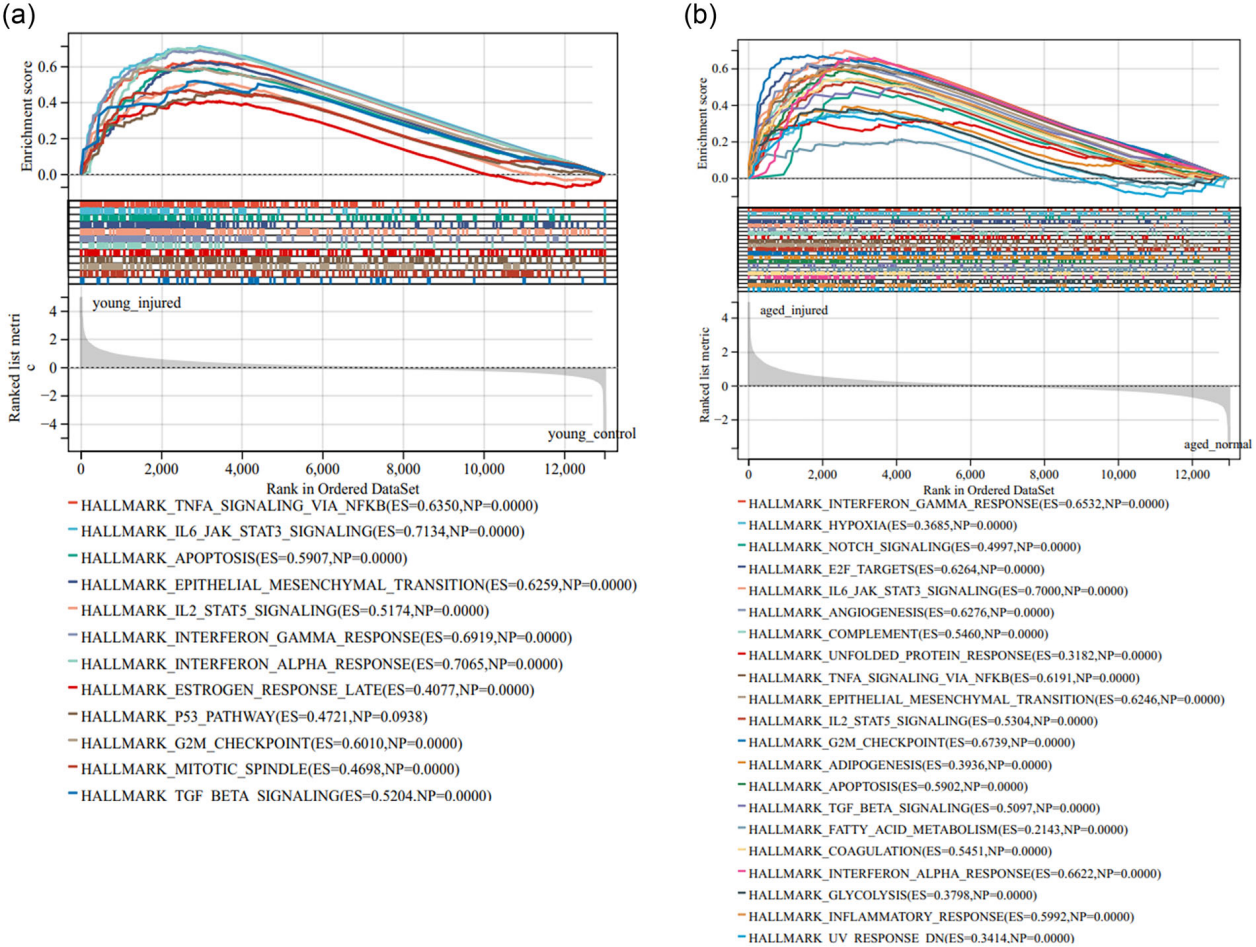
**Enriched signaling pathways in the young and aged SCI groups**. GSEA was performed to explore the enriched signaling pathways in the (a) young and (b) aged SCI groups.

Based on the GSEA results, the enriched signaling pathways in the two SCI groups were probed. A total of 12 signaling pathways were exhibited in the young SCI group, including TNFA signaling via NF‐κB, IL‐6/JAK/STAT3 signaling, apoptosis signaling, epithelial‐mesenchymal transition (EMT) pathway, IL2/STAT5 signaling, interferon (IFN) gamma response, IFN alpha response, estrogen response late, p53 pathway, G2M checkpoint, mitotic spindle signaling, and TGF beta signaling (Figure 2a). For the aged SCI group, a total of 21 enriched signaling pathways were discovered, including IFN gamma response, hypoxia, Notch signaling, E2F targets, IL‐6/JAK/STAT3 signaling, angiogenesis, complement, unfolded protein response, TNFA signaling via NF‐κB, EMT pathway, IL2/STAT5 signaling, G2M checkpoint, adipogenesis, apoptosis, TGF beta signaling, fatty acid metabolism, coagulation, IFN alpha response, glycolysis, inflammatory response, and UV response DN (Figure 2b).

### 3.3 Identification of hub genes based on WGCNA

**FIGURE 3:**
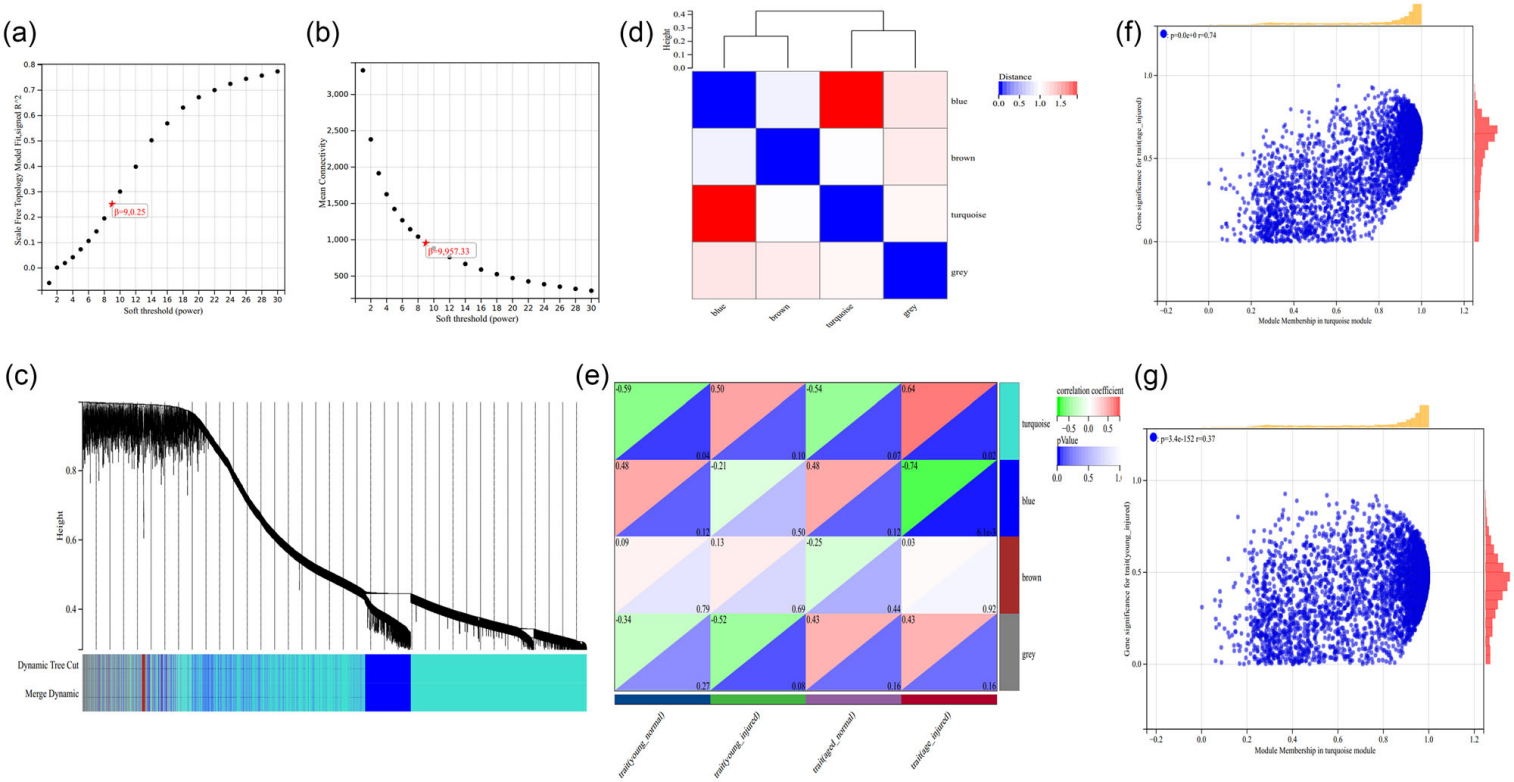
**Identification of hub genes based on WGCNA**. (a) The scale‐free index for various soft‐threshold powers (β). (b) The mean connectivity for various soft‐threshold powers. (c) Dendrogram of all DEGs clustered based on the measurement of dissimilarity (1‐TOM). (d) Cluster of module traits. (e) A heatmap was used to present the correlation between the module and clinical traits of SCI mice (young, aged). Scatter plot of the gene significance and MM in the turquoise module in (f) aged or (g) young SCI mice.

The selection of hub genes was performed under the conditions of |MM| > 0.9, GS > 0.2, and weight > 0.1. The gene cluster results of GSE93561 samples were explored using Pearson's correlation coefficient. The soft‐threshold power in WGCNA was determined to be 9 to construct a scale‐free network (Figure 3a&b). A total of three modules were identified using average hierarchical clustering as well as dynamic tree clipping (Figure 3c), including blue (1082 genes), brown (64 genes), and turquoise modules (4594 genes), and the turquoise module was positively correlated with the blue module (Figure 3d). Moreover, based on the heatmap of modules and phenotypes (Figure 3e), the turquoise module was found to be most significantly correlated with the phenotypes of the aged injured (*r* = .74, *p* < .05) and young injured groups (*r* = .37, *p* < .05) (Figure 3f & g). Subsequently, 3181 hub genes were obtained after screening the key turquoise modules.

### 3.4 Important genes and corresponding pathways in SCI

**FIGURE 4:**
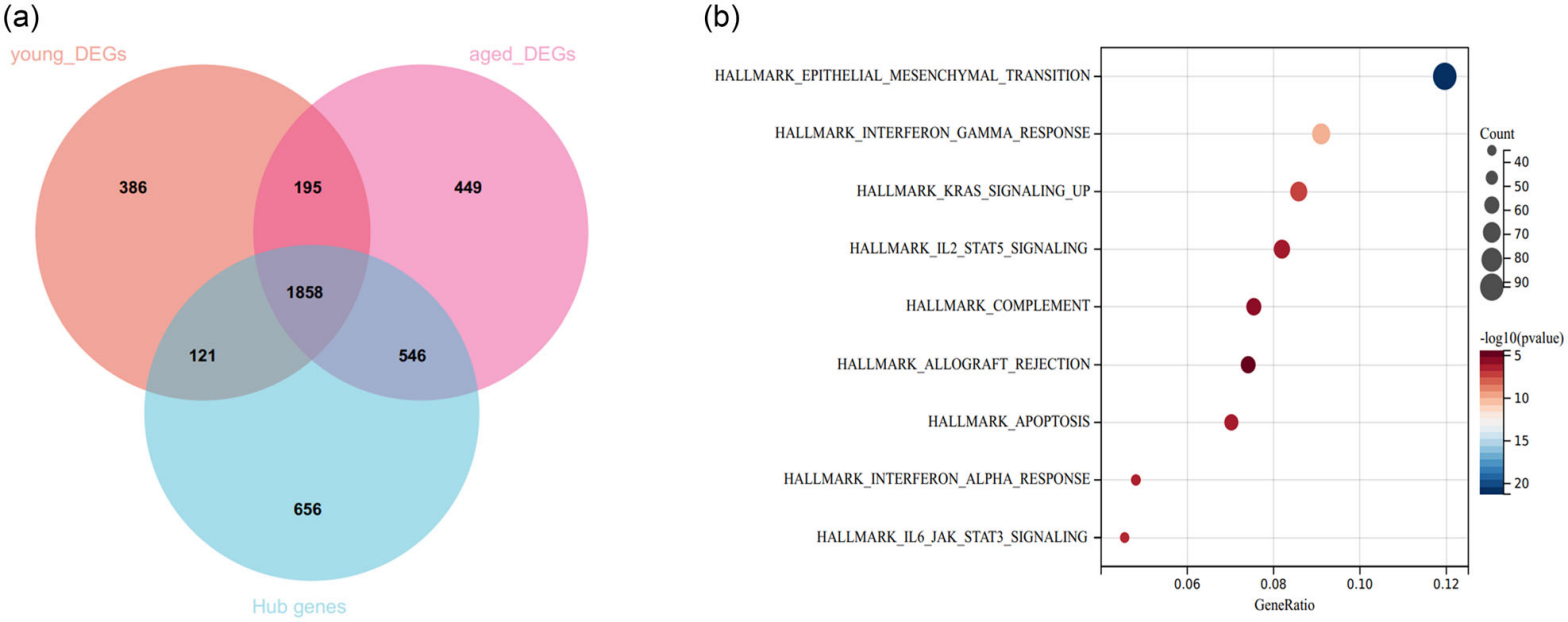
**Identification of important genes and corresponding signaling pathways in SCI**. (a) Venn diagram of the intersection of DEGs in young and aged SCI mice and the hub genes obtained from the WGNCA analysis. (b) Nine signaling pathways were screened using KEGG enrichment analysis under FDR < .1 and *p* < .05.

The important genes in SCI were obtained by intersecting the DEGs in the young and aged SCI mouse groups with the hub genes selected based on WGNCA analysis. As revealed by the Venn diagram in Figure 4a, a total of 1858 intersecting genes were screened (Figure 4a). Then, KEGG enrichment analysis was performed to explore the potential signaling pathways under the conditions of FDR < 1 and *p* < .05, and nine enrichment signaling pathways, including EMT, IFN gamma response, KARS signaling, IL2/STAT5 pathway, IL6/JAK/STAT3 pathway, allograft rejection, complement, apoptosis, and IFN alpha response, were observed (Figure 4b).

## 4 DISCUSSION

Aging is an important factor involved in regeneration after CNS injury (Bar‐Or & Antel, 2016). It has been reported that the levels of proinflammatory cytokines and chemokines are significantly lower in young mice than in adult mice after SCI (Kumamaru et al., 2012). In this study, we explored potential biomarkers in young or aged SCI mice based on the GEO dataset through bioinformatics analysis. We found 2560 DEGs in the young SCI group, which included 1733 highly expressed and 827 lowly expressed RNAs. For the aged SCI group, we found a total of 3048 DEGs including 1856 upregulated and 1192 downregulated RNAs. The enriched signaling pathways as well as the hub genes in the young or aged SCI groups were also analyzed. Finally, the DEGs in the aged and young SCI groups intersected with the hub genes, and we obtained 1858 genes by intersection. KEGG enrichment analysis revealed nine enriched signaling pathways of these important genes.

As reported previously, a variety of signaling pathways are implicated in SCI such as the NF‐kB (H. Liu et al., 2021), AMPK‐mTOR‐TFEB signaling (C. Wu et al., 2021), and PI3K/AKT pathways (Xu et al., 2020). Therefore, targeting these signaling pathways may provide promising insight for SCI treatment. Here, GSEA was performed to explore the enriched signaling pathways in the young or SCI groups. It possesses many advantages relative to single‐gene methods. GSEA simplifies the interpretation of large‐scale experiments by determining pathways by focusing on gene sets rather than on high‐scoring genes. The young SCI group was mainly associated with 12 signaling pathways, such as IL6/JAK/STAT3 signaling, IFN alpha response, EMT pathway, and TNFA signaling via NF‐kB. The aged SCI group was suggested to be closely correlated with IL6/JAK/STAT3 signaling, IFN gamma response, angiogenesis, E2F targets, IFN alpha response, and G2M checkpoint. Previous studies have revealed that IL‐6 mainly induces early JAK/STAT3 activation in spinal microglia and facilitates neuropathic pain development (Dominguez et al., 2008; Yamauchi et al., 2006). In addition, it has been reported that CNS injury is accompanied by the EMT pathway (Vivinetto et al., 2020). After SCI, the inflammatory response promotes the production of proinflammatory factors, such as TNF‐α and IL‐6, leading to suppressive effects on the regeneration of neurites (Schwartz et al., 1999). The activation of NF‐κB signaling further elevates proinflammatory factor levels and contributes to neuronal death (J. Chen et al., 2017). The IFN response pathway is reported to be critically implicated in traumatic brain injury and SCI (Roselli et al., 2018). IFN‐α, IFN‐β, IFN‐ε, IFN‐κ, and IFN‐ω are type‐I IFNs, and IFN‐γ belongs to type‐II IFNs (McNab et al., 2015). Type‐I signaling inhibition leads to a significant reduction in proinflammatory IL‐1β and IL‐6 levels and an increase in the anti‐inflammatory mediator IL‐10, which suggests an inhibited inflammatory response after brain injury (Karve et al., 2016). IFN‐γ signaling plays a critical role in SCI and may exert protective or detrimental effects after brain injury, which probably depends on the cellular source and the released cytokine concentration (Fujiyoshi et al., 2010; Sun et al., 2018). Angiogenesis post‐SCI is also a research focus that attracts increasing attention. SCI induces immediate nervous tissue disruption and direct vessel injury, which results in neuronal death (Figley et al., 2014). Regeneration of axons is revealed to grow along vessels, and aberrant vessel regeneration may inhibit post‐SCI repair (Rauch et al., 2009). Thus, postinjury angiogenesis is suggested as a potential therapeutic target for SCI (Cao et al., 2021; Yao et al., 2021). E2F is critically involved in cellular senescence (Narita et al., 2003), and E2F1 activation after brain injury is suggested to play a role in neuronal apoptosis following traumatic brain injury (Aubrecht et al., 2018; W. Liu et al., 2013). In addition, previous studies have also performed bioinformatics analysis of key signaling pathways in SCI (J. H. Li et al., 2020). A study proposed by (Z. Li et al., 2022) noted that the TLR4/MyD88/NF‐κB inflammatory pathway was activated in SCI through bioinformatics analysis, which further supports our findings. The WGCNA method was used for the construction of a co‐expression network, and modules with clinical features were determined (Tian et al., 2020). In total, three modules were identified, with 1082 RNAs for the blue modules, 64 RNAs for the brown modules, and 4594 RNAs for the turquoise modules. The turquoise module was identified to be positively correlated with the blue module and most significantly associated with the phenotypes of the aged injured and young injured groups. With the unique soft threshold algorithm of WGCNA, the gene expression network is distributed with a free‐scale network, which indicates the higher reliability of our results. Finally, the important SCI genes were screened by intersecting the DEGs in the young and aged SCI groups with the hub genes from the WGNCA analysis. In total, 1858 genes were selected by intersection, and KEGG enrichment analysis revealed nine enrichment signaling pathways of these important genes such as the EMT process, IFN gamma response, and KRAS signaling. In line with our study, Q. Chen et al. (2021) also used WGCNA to detect gene expression in SCI and found that six genes may serve as a potential prognostic target for the diagnosis and treatment of SCI.

However, the limitation of our study was that the limited data published in the open dataset and the incomprehensive clinicopathological features analyzed may lead to potential statistical bias. Therefore, a more comprehensive study should be conducted in the future to identify more biomarkers for SCI treatment.

In conclusion, we identified DEGs, enriched signaling pathways, and hub genes in the young and aged SCI groups. By intersecting the DEGs and the hub genes, 1858 important genes and the related signaling pathways were screened out. The findings of this study may provide insight into targeted therapy for SCI.

## AUTHOR CONTRIBUTIONS

**Wei Miao**: Conceptualization; data curation; formal analysis; investigation; methodology; software; validation; visualization; writing—original draft. **Zheng Su**: Conceptualization; data curation; formal analysis; investigation; methodology; validation; writing—review and editing. **Huilin Cheng**: Funding acquisition; project administration; resources; supervision; writing—review and editing.

## CONFLICT OF INTEREST STATEMENT

There are no conflict of interest in this study.

### PEER REVIEW

The peer review history for this article is available at https://publons.com/publon/10.1002/brb3.3293

## Data Availability

Original data of this study can be obtained from the corresponding author upon reasonable request.
